# Analysis of Wild Type LbCpf1 Protein, and PAM Recognition Variants, in a Cellular Context

**DOI:** 10.3389/fgene.2020.571591

**Published:** 2021-01-07

**Authors:** Ujin Shin, Vincent Brondani

**Affiliations:** Center for Genome Engineering, Institute for Basic Science, Daejeon, South Korea

**Keywords:** Cpf1, genome engineering, PAM, specificity, selectivity

## Abstract

Nucleases used in genome engineering induce hydrolysis of DNA phosphate backbone in a sequence-specific manner. So far CRISPR-Cas, the RNA-guided nucleases, is the most advanced genome engineering system. The CRISPR nucleases allows recognition of a particular genomic sequence with two distinct molecular interactions: first, by direct interaction between the nuclease and the protospacer-adjacent motif, wherein discrete amino acids interact with DNA base pairs; and second, by hybridization of the guide RNA with the target DNA sequence. Here we report the application of the single strand annealing cellular assay to analyze and quantify nuclease activity of wild type and mutant CRISPR-Cpf1. Using this heterologous marker system based on GFP activity, we observed a comparable PAM recognition selectivity with the NGS analysis. The heterologous marker system has revealed that LbCpf1 is a more specific nuclease than AsCpf1 in a cellular context. We controlled the *in vitro* activity of the Cpf1 nuclease complexes expressed in mammalian cells and demonstrated that they are responsible of the DNA cleavage at the target site. In addition, we generated and tested LbCpf1 variants with several combinations of mutations at the PAM-recognition positions G532, K538 and Y542. Finally, we showed that the results of the *in vitro* DNA cleavage assay with the wild type and mutants LbCpf1 corroborate with the selection of 6TG resistant cells associated to the genomic disruption of *hprt* gene.

## Introduction

For reverse genetics approaches, genomic modification is often required to establish the functional link between an observed phenotype and a particular gene. Precise DNA modification such as base substitution or introducing insertion/deletion are crucial for those functional validation. Indeed, generating nucleotide substitutions (point mutations) or indels (insertions/deletions) can lead to gene knockout by generating a premature stop codon or to truncated gene products by frameshift mutation. Various strategies have been developed to induce genetic alterations, and the resulting cells harboring desired changes are suitable for phenotypic analyses. In some cases, genetically modified cells or organisms can be isolated from the population upon a positive selection pressure. The protein Hypoxanthine Phosphoribosyl transferase (HPRT) is an enzyme that catalyzes conversion of hypoxanthine to inosine monophosphate, and guanine to guanosine monophosphate. The protein is encoded by a unique gene (*hprt*) in human cells, carried by the chromosome X. Disruption of the *hprt* gene allows a survival of the mutated cells upon treatment with the cytotoxic chemical agent 6-Thio-Guanine (6-TG) (Fenwick and Caskey, 1975), thus leading to positive selection of *hprt* homozygote -/- cells in cultures.

In recent years, the zinc finger nucleases (ZFNs) (Bibikova et al., 2001), the transcription activator-like effector nucleases (TALENs) (Christian et al., 2010), and more recently, the bacterial adaptive CRISPR-Cas systems (Gasiunas et al., 2012; Jinek et al., 2012) were efficiently established to generate eukaryotic mutant cells. Those engineered nuclease enzymes induce DNA double strand break (DSB) at genomic target sites. The CRISPR-Cas system has been developed to perform specific genetic modifications by generating precise DSB in the targeted genomic locus. Unlike ZFNs and TALENs that fuses DNA-binding domains to the DNA cleavage domain from the *FokI* restriction endonuclease, the CRISPR-Cas system require a guide RNA that directs the nuclease to the target DNA sequence. The ribonucleoprotein (RNP) complex catalyze the DNA cleavage at target sites. Since DNA is targeted by specific base-pairing with the guide RNA, the CRISPR-Cas system is highly versatile and more specific for the binding to target DNA sequences within the genome (Kim et al., 2015; Yan et al., 2017). More recently, base editors have been developed by fusing Cas9 nickase D10A variant with cytosine deaminase, or engineered adenine deaminase domain. Each fused protein, termed Cytosine Base Editor (CBE) and Adenosine Base Editor (ABE), are enabled to catalyze the conversion of C-G to T-A and A-T to G-C base pairs, in a sequence specific manner (Komor et al., 2016; Gaudelli et al., 2017).

**FIGURE 1:**
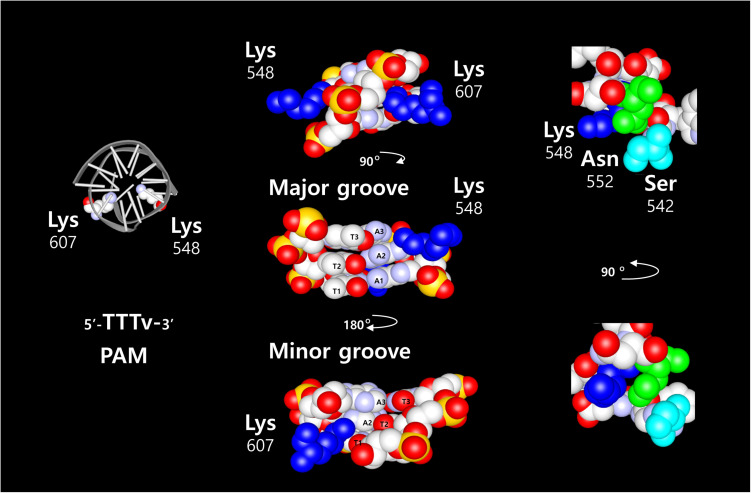
Graphic representation of AsCpf1 amino acids in complex with its DNA sequence. On the left side, schematic structure of the DNA with the two Lysine 548 and 607 required for the AsCpf1 5′-TTTv-3′ PAM sequence recognition. The three T-A base pairs are represented with the two Lysine 548 and 607 (blue) to show the direct contacts between the second Adenine base and the two Lysine. The contact occurs from both side of the DNA helix. Lysine 548 on the major groove and Lysine 607 on the minor groove. Other amino acids in vicinity to the lysine 548 (Asparagine 552: green, and serine 542: light blue), exposed on the major groove side of the DNA, are depicted in the picture on the right side.

Two of the most common nucleases for CRISPR-Cas systems, SpCas9 and Cpf1 (also named Cas12a) proteins (Zetsche et al., 2015), share similar features when associated with their respective guide RNA. However, they differ in structural organization and DNA cleavage properties. Cpf1 only requires a crRNA to bring the specificity for a target in the RNP complex, whereas SpCas9 requires additional trans-activating RNA (tracrRNA) as well, that bound to the protein for its stabilization. This was simplified by fusing the crRNA and tracrRNA into a chimeric RNA called single guide RNA (sgRNA) (Jinek et al., 2012). In addition to the DNA sequence recognized by the guide RNA, the CRISPR proteins interact with a short DNA region adjacent to the target DNA sequence, termed as Protospacer Adjacent Motif (PAM). The PAM recognized by SpCas9 is 5′-NGG-3′ and is located downstream to the target DNA sequence. The sequence recognized by Acidaminococcus (As) and Lachnospiraceae bacterium (Lb) Cpf1 is 5′-TTTv-3′ and is located upstream of the target DNA sequence. Nevertheless, those two CPF1 proteins have a different specificity and selectivity for the PAM sequence. In addition, the DNA cleavage site itself is different between those complexes, the SpCas9 cleavage site is proximal to the PAM sequence and inside of the nuclease structure. In contrast, the cleavage site of the Cpf1 nucleases complexes is distal to the PAM sequence, and outside of the core structure. The DSB induced by SpCas9 results in blunt ends, whereas Cpf1 generates 5′ overhangs end. The target specificity of SpCas9 and Cpf1 proteins are determined by the crRNA sequence, and the molecular interaction between few amino acids from the PAM Interacting Domain (PID) and the PAM sequence. The crystal structure of SpCas9 (Anders et al., 2014; Nishimasu et al., 2014) in complex with the sgRNA and the target DNA revealed two arginine residues that are directly in contact with the two guanines of the PAM sequence on the major groove side of the DNA. This interaction involves hydrogen bonds that take place on the major groove side of the DNA. By comparison, crystal structure of AsCpf1 (Yamano et al., 2016) in complex with the crRNA and target DNA shows that the PAM sequence recognition requires two lysines (Figure1: residues K548 and K607). The two lysine are interacting with the second complementary base (Adenine) of the 5′-TTTv-3′ sequence. Both lysines are in contact from both sides of the double helix, K548 on the major groove side, and K607 on the minor groove side. Two amino acids, N552 and S542, are near the residue K548 and structurally in the vicinity of the DNA. Those amino acids are interacting with the phosphates from the DNA backbone. In LbCpf1 structure, the PAM/PID interaction is similar (Lysines K538 and K595) to the AsCpf1, but the amino acids in vicinity with the DNA backbone are different (G532, Y542). Because of the structural features of Cpf1 proteins, we focused our research on the properties of those proteins in a cellular context.

In this report, we used two strategies to analyze the nuclease activities in eukaryotic cells. First, the genomic modifications were measured by the indels frequency at the target site, which are generated by the non-homologous end joining (NHEJ), using the deep sequencing approach. Secondly, the single-strand annealing (SSA) based on a plasmid harboring the nuclease target site that is between two fragments of the reporter gene with repeated sequences. Inducing DSB at the target site, and the subsequent recombination of the repeated sequences by homology-directed recombination (HR), results in the restoration of a functional reporter gene. Thus, the nuclease activity in the cells is measured by the signal from the heterologous reporter gene (Cathomen et al., 2008; Tóth et al., 2016).

The study is investigating the PAM recognition of Cpf1 proteins in a cellular context using deep sequencing with a crRNA collection that target the exon 3 of *hprt* gene. We focused our tests on the variation of two nucleotides from the PAM sequence (position -2 and -3, 5′-TNNa-3′) that are common to all Cpf1 proteins orthologues. Thereby each selected target sequences requires one of the 16 possible 5′-TNNa-3′ sequence. A 16 pGFP-SSA plasmid library was also designed with the same DNA target sequence but flanked with the different 5′-TNNa-3′ PAM. The indels frequencies observed with AsCPF1 and LbCPF1 are consistent with the SSA assay results from the pGFP-SSA library. In addition, we controlled the *in vitro* nucleases activity with the purified Cpf1 complexes from mammalian cells. We designed, and tested LbCpf1 mutations at position 532, 538 and 542 to modulate the PAM recognition. Using the SSA assay, we observed that the mutant LbCpf1-NS has the same PAM specificity and selectivity than AsCpf1. We found that the mutants LbCpf1 RAR and RSR recognize the three sequences 5′-TTTa-3′, 5′-TATa-3′ and 5′-TACa-3′. Those mutants are suitable to perform the *in vitro* cleavage of DNA substrates with the corresponding PAM sequences. Finally, we controlled their efficacy to target genomic sites, by disrupting the *hprt* gene (selection of 6-TG resistant cells), with guides crRNA associated to the three different types of PAM sequences at the genomic sites.

## Materials and Methods

### Cell Culture

HeLa (ATCC CCL-2) and HEK 293 (ATCC CRL-1573) cells were cultivated at 37 °C, 5 % CO2 in Dulbecco’s modified eagle’s medium containing 4.5 mg/L D-glucose (DMEM, WelGene, South Korea), Glutamine 2,5 mM, supplemented with 10 % heat inactivated fetal bovine serum (FBS, Gibco, United States) and antibiotics solution (100 U/ml penicillin and 100 μg/mL streptomycin solution, Gibco, United States). Cells were frozen at the density of 1 × 10^7^ cells per mL in DMEM containing 50 % FBS and 10 % dimethyl sulfoxide (DMSO, Sigma-Aldrich, United States). For Isolation of *hprt* −/− cells, the transfected cells were incubated for 4 days with the 6TG (Sigma-Aldrich, United States) at 20 μM final concentration. In order to remove the product of the target gene insight the cells, a minimum of 5 days incubation is required prior the selection with the 6TG. For shorter incubation time, only the nuclease effects on the target DNA can be elucidated. To visualize the cells, the cultures were washed and stained using methylene blue and inspected by microscopy (Pimovert Zeiss, Germany).

### Plasmids and Guide RNA

For the design of the target DNA used in this study, the crRNA sequence was established to target the human exon 3 *hprt* gene was selected among a collection of guide sequences (data not shown) and demonstrated a high genomic modification ability. The expression plasmid for the crRNA LbCpf1 was transfected into Hela cells, after 5 days exposure and cell culture, the cells were selected using the 6TG and analyzed by Deep sequencing (See Supplementary Data 1). A *dnmt1* crRNA target sequence was used as a negative control to confirm the specificity. All the *hprt* gene target sequences used in this study were cloned in the U6 promoter-driven RNA expression vector pU6-As-crRNA and pU6-Lb-crRNA for AsCpf1 and LbCpf1, respectively (The oligonucleotide sequences cloned are reported in Supplementary Data 2).

The pGFP-SSA plasmids were generated by cloning of two *gfp* gene fragments containing the left- and right-repeats (LR and RR, respectively: Supplementary Data 3A,B) into pcDNA3 vector. The SSA target DNA fragments, with the corresponding PAM sequences, were inserted between the LR and RR using *EcoRI* and *BamHI* restriction sites. An adenine was placed at the first position of the PAM sequence adjacent to the target sequence and described as 5′-TNNa-3′ (The 5′-TNNa-3′ sequences cloned in the pGFP-SSA constructs are reported, Supplementary Data 4). The assay was optimized, and controlled, by comparison of the Hela cells transfection with the full length *gfp* and the *EcoRI* linearized SSA plasmids (Supplementary Data 5A). In order to reduce the background signal in the experiments, the plasmids were purified using anion-exchange silicate resin (Macherey-Nagel, Germany) with bacteria cell cultures at low turbidity (Exponential phase) to prevent the presence of linearized plasmid DNA in the samples. The DNA concentration was characterized by spectrometry (OD260 nm) and controlled by double DNA digest with restriction enzymes and agarose gel stained with ethidium bromide. The quality and the quantity of DNA in the samples were compared by intensity of the bands in the agarose gel. All the DNA sequences used in this study, that target *hprt* exon 3, were tested for their activity by co-transfection with the corresponding 5′-TTTa-3′ PAM pGFP-SSA target reporter and the Wt LbCpf1 protein (Supplementary Data 6).

The expression plasmids of human codon-optimized AsCpf1 (pCDNA3-AsCpf1) and LbCpf1 (pCDNA3-LbCpf1) were prepared according to previously reported study (Tóth et al., 2018). The plasmid expressing AsCPF1-MBP and LbCPF1-MBP fusion proteins, the MBP tag coding cDNA was cloned, at the C terminal part of *Cpf1* genes, between *BamHI* and *EcoRI* restriction sites. As described (Bokhove et al., 2016), the MBP tag C terminal fusion allow the purification of full-length proteins in a single step.

LbCpf1 PAM mutants were generated using QuickChange II Site directed Mutagenesis Kit (Agilent, United States) according to manufacturer’s instructions. The oligonucleotides used for mutagenesis are reported in Supplementary Data 4.

### SSA Assay

Cryopreserved HeLa cells were thawed and reconstituted in DMEM with 10 % FBS at a density of 5 × 10^5^ cells per mL, then plated in 24 well plate (1 mL per well) 4 h prior to transfection. Transfection was performed using Lipofectamine 2000 (Invitrogen, United States) under the following conditions: 5 μL Lipofectamine 2000 (1 mg/mL), 300 ng of Cpf1 expression vector, 500 ng of the crRNA plasmid, 500 ng of corresponding pGFP-SSA target construct, were mixed in OptiMEM I (Gibco, United States) to a final volume of 500 μL. The pGFP-SSA 5′-TNNa-3′ PAM plasmid library experiments were performed with a lower quantity of nuclease expression vector (100 ng). After 2 days incubation, image of fluorescent cells, expressing GFP protein, were obtained using a Nikon Eclipse confocal microscope (Nikon, Japan). The cell surviving was measured using Cell-titer-Glo luciferase assay (Promega, United States). The fluorescence was characterized using a Filter max F5 reader Multimode (Molecular Device, United States). For each transfection experiment a pGFP construct was co-transfected under the same conditions as a GFP signal stability control, and a pGFP-SSA *EcoRI* linearized as a reference for the percentage of fluorescence calculation. Duplicates or triplicates transfection were performed during the same experiment for a better quantitative reproducibility.

### Deep Sequencing Analysis

Hela cells were seeded (5 × 10^5^ cells/well) in a 24 well plate and were transfected with plasmids expressing crRNA and Cpf1 Wt or mutants (500 ng of each vectors) using lipofectamine 2000 (Invitrogen, United States), according to the manufacturer instructions. Following 3 days exposure and incubation, the cells were collected, and genomic DNA isolated using DNeasy Blood and Tissue kit (Quiagen, Netherlands). The genomic region, encompassing the *hprt* exon3 target sites was amplified using the following primers: NGS1-Fw (5′- CAAGGTCTTGCTCTATTGTCCAG-3′) and NGS1-rev (5′-CCCTTGAGGACACAGAGG-3′). The amplified fragments were analyzed by deep sequencing, according the previously published experimental procedure (Kim et al., 2016; Tóth et al., 2018).

### Protein Expression and *in vitro* Nuclease Activity

HEK293 cells were cultivated in a 10 cm dish at 80% confluency and were transfected using lipofectamine 2000 reagent (100 μL) with 50 μg pcDNA3-AsCPF1-MBP or pcDNA3-LbCPF1-MBP, with or without 10 μg of the pU6 guide RNA expression vector. 48 h post transfection, the cells were collected, and Cpf1-MBP fusion protein were purified with amylose resin (New England Biolabs, United States). The cells were washed with PBS and re-suspended in 500 μL of Lysis buffer containing Tris-HCl pH8 100 mM, KCl 50 mM, MgCl2 20 mM, Triton x-100 0.1%, RNase Inhibitor (200 Unit/mL: New England Biolabs, United States) and Protease inhibitor minus EDTA (Merk, United States). The cells were broken by thermal shock (freezing in liquid nitrogen and thawing at 42°C). After centrifugation for 10 min at 15000 rpm, the supernatants containing the recombinant proteins were incubated for 2 h with amylose beads (150 μL). The beads were washed three times with 1 mL of lysis buffer, the recombinant proteins were eluted with a Lysis buffer adjusted at 20 mM maltose (Merk, United States). The *in vitro* cleavage assay was performed in lysis buffer, using *XmaI* linearized pGFP-SSA target vector as a substrate DNA. After 15 min incubation at 37°C, the reactions were arrested by addition of EDTA, proteinase K, and incubated at 60°C for 10 min. The DNA fragments were analyzed on a 1% agarose gel with ethidium bromide.

## Results

### Analysis of Cpf1 Proteins PAM Selectivity Using Deep Sequencing at Genomic Target Sites

**FIGURE 2:**
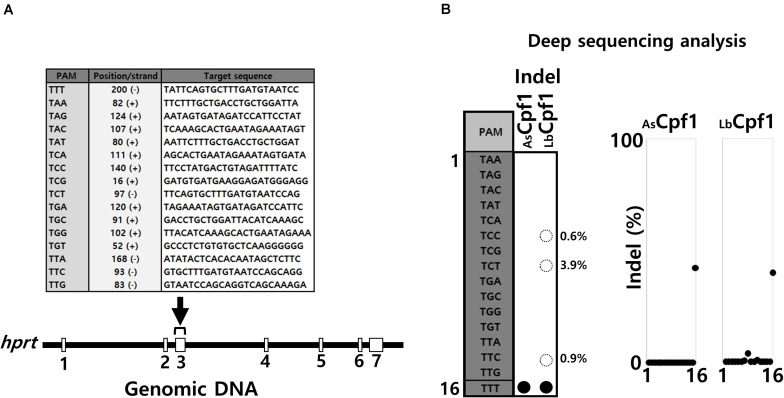
Comparison of AsCPF1 and LbCpf1 PAM recognition by Deep sequencing analysis. **(A)** Target sequences: The sequences of the target *hprt* gene (exon3) are depicted in the table with the corresponding flanked 5′-TNNa-3′ sequences at the genomic sites. **(B)** NGS analysis: Deep sequencing analysis and indels frequency (%) observed for each guide RNA by AsCPF1 and LbCpf1 protein co-expression. The table on the right side summarize the Graph of the activity with the 16 guide RNA/5′TNNa-3′ for each protein. The expression vectors quantity was adjusted for both enzyme (AsCPF1 and LbCpf1) to obtain the same indels frequency with the optimal 5′-TTTa-3′guide RNA.

We first investigated the Cpf1 proteins PAM selectivity in a cellular context by analyzing the activity of several crRNA with different PAM sequences that target the same genomic area corresponding to the *hprt* exon 3. We used deep sequencing analysis to quantify indels frequencies that reflect the nuclease activity at the genomic target sites. The Cpf1 proteins need to interact with the PAM adjacent sequence to perform the DNA cleavage. Based on the protein/DNA interactions described in Cpf1 complexes resolved structures, we decided to select 16 different endogenous target sequences (Figure 2A) that are flanked by one of each different permutation of 5′-TNNa-3′ PAM sequences. Hela cells were transfected with AsCpf1 and LbCpf1 expression vectors, together with each crRNA expression plasmids. Following genomic DNA isolation for each sample, indels frequencies induced by the different crRNA were determined by deep sequencing analysis for an identical PCR amplicon. The crRNA targeting the site flanked by the PAM 5′-TTTa-3′ sequence was used as a control, to verify that the activity of AsCpf1 and LbCpf1 are similar under our experimental conditions. The results depicted in Figure 2B show the quantification diagram (right panel) and a table summarizing the activity observed with each crRNA (left panel). For both proteins, AsCpf1 and LbCpf1 with the PAM 5′-TTTa-3′ crRNA, the indels frequencies detected are around 40%. All the other crRNAs, analyzed under AsCpf1 protein expression are not able to induce indels at the corresponding target sites, whereas LbCpf1 when combined with crRNA targeting the sequences flanked by the PAM 5′-TCTa-3′, 5′-TTCa-3′ and 5′-TCCa-3′ showed some nuclease activities, represented by low indels frequencies.

### Analysis of Cpf1 Proteins Activity Using SSA Assay

**FIGURE 3:**
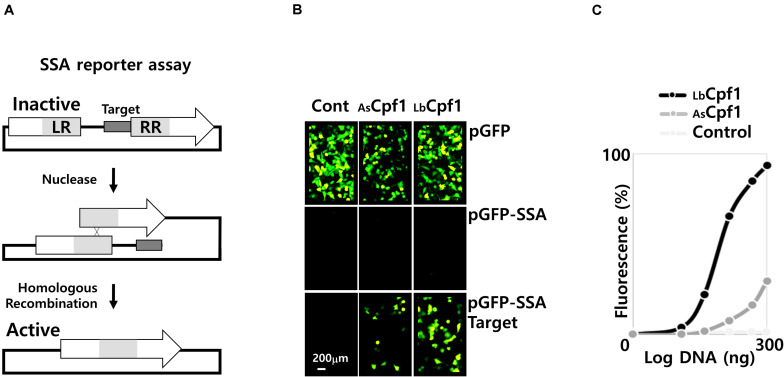
SSA assay. **(A)** Representation of the SSA assay: The two fragments of the inactive reporter gene are represented with the nuclease target site in between. The left and the right repeated sequence are depicted as LR and RR, respectively. Following the nuclease cleavage, at the target site, the fragment is recombined to reconstitute a full-length active reporter gene. **(B)** Pictures of cells using a fluorescent microscope (×20): Fluorescent cells were inspected after transfection and 48 h incubation. Three reporter constructs were transfected with the nucleases, the pGFP native expression vector to control the GFP signal stability, the negative control pGFP-SSA vector without target site, and the pGFP-SSA 5′-TTTa-3′PAM target plasmid. The control panels (Cont.) on the left, are reporter vectors transfected without nucleases. **(C)** Dose response curve of the AsCPF1 and LbCPF1: Cpf1 expression vectors were co-transfected with the guide expression vector and the pGFP-SSA 5′-TTTa-3′PAM reporter construct. A negative control was performed with LbCpf1 and guide expression vector against the *dnmt1* gene.

We next investigated the usage of the SSA assay with a plasmid to assess the activity of nuclease in a cellular context. We used the GFP reporter gene as a readout marker. This strategy is based on the reconstitution, by homologous recombination (HR), of a functional full-length *gfp* gene. The *gfp* reporter gene is activated because of the cleavage of the plasmid by a nuclease at a specific target site. The target site from *hprt* gene was inserted between two inactive fragments with a repeated sequence (Figure 3A). After DNA cleavage, the repeated sequence recombines by HR, thus generating an active gene. This cellular assay allows to analyze the cellular activity of a nuclease upon its co-transfection with the reporter plasmids. Nevertheless, this artificial system reflects a comprehensive nuclease activity if the enzyme is highly specific, and sufficiently selective, based on the fact that unspecific nuclease activities are generating multiple cleavages of the plasmid, and leads to the degradation of the reporter construct. This in turn abolish the reporter gene signal and, non-specific cleavage is generating false negative samples in the SSA analysis. Thus, for a short time of incubation (48 h) after the plasmid transfection, the destabilization of the GFP signal suggest that the nuclease as a high non-specific DNA cleavage activity that leads to the reporter plasmid degradation.

We analyzed the activity of Cpf1 proteins, by co-transfection of the nuclease expressing plasmids (300 ng DNA of CRISPR protein expression vectors) with the corresponding crRNA expression plasmids, and reporter vector. The experiment was performed with the pGFP control, or the pGFP-SSA construct with or without a specific target sites flanked by the optimal 5′TTTv-3′ PAM sequence (Figure 3B). Without co-transfection of a nuclease (Figure 3B, Cont. Panels), only the pGFP reporter vector show fluorescent cells. Indeed, the pGFP SSA vectors require a plasmid linearization between the repeated sequences to induce GFP expression (Supplementary Data 5A, transfection control with a circular and an *EcoRI* linearized pGFP-SSA vector). The pGFP control experiment (upper panels) was performed to observe the stability of the fluorescent signal upon nuclease expression. The result shows that the number of fluorescent cells decreased by AsCpf1 nuclease co-transfection, indicating that the GFP signal is destabilized, whereas the GFP expression remains similar to the GFP control with LbCpf1 (Figure 3B). The quantification (Supplementary Data 5B, quantification of GFP signal and number of cells) indicated that the nuclease expression did not significantly affect the cell surviving, but the GFP fluorescent signal is dramatically reduced with the AsCpf1 nuclease and crRNA guide expression. The pGFP-SSA target plasmids (Figure 3B, lower panels) show fluorescent cells with all nucleases tested, whereas no cells are GFP positive with the plasmid construct without target site (middle panels). The results are demonstrating the requirement of the target site, between the repeated sequences, to induce GFP expression. The GFP positive cells are more abundant with LbCpf1 than with AsCpf1, suggesting that the GFP signal destabilization occur with the pGFP-SSA vector and AsCpf1, which is consistent with the case of pGFP and AsCpf1. The Figure 3C show the quantification, and the dose response curves, obtained with both nucleases targeting the same *hprt* sequence, and a negative control corresponding to LbCPf1 co-transfected with a non-specific crRNA targeting *dnmt1* gene. The LbCpf1 nuclease exerts a better SSA activity than AsCPf1. To compare the SSA activity of the Cpf1 nucleases with spCas9, we also analyzed the spCas9 wild type and nickase D10A activity (Supplementary Data 7). The results demonstrated that spCas9 has a similar activity to AsCpf1 and show that the Nickase D10A, that require an inverted tandem repeat, is more active than the Wt SpCas9 and do not destabilize the GFP signal in the control experiment.

**FIGURE 4:**
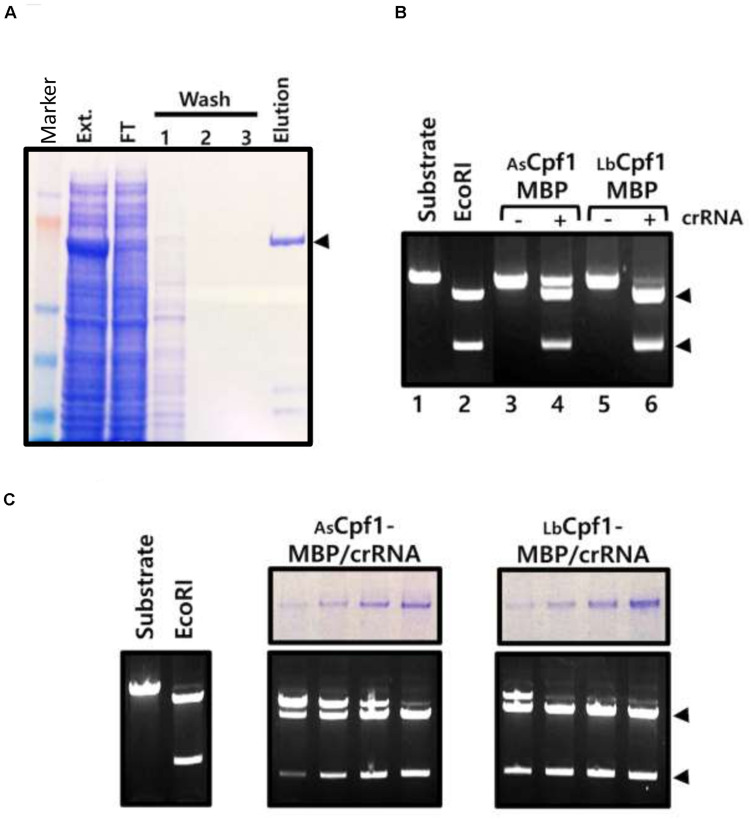
*In vitro* activity of cellular Cpf1 proteins: **(A)** AsCpf1-MBP protein purification: The HEK293 AsCpf1-MBP cellular extract (Ext.) was analyzed on an 8% denaturing acrylamide gel, together with the Flow through (F.T.), washing steps (1, 2, and 3), and Elution of the affinity chromatography with amylose beads. The black triangle indicates the position of the migration of the AsCpf1-MBP protein (170kDa). **(B)** *In vitro* activity of Cpf1-MBP purified proteins: Cleavage activity of Cpf1-MBP proteins expressed or not with the crRNA was performed with pGFP-SSA 5′-TTTa-3′ linearized *XmaI* target plasmid. The plasmid substrate was digested with the *EcoRI* restriction enzyme to control the size of the products. **(C)** Dose response activity of the purified Cpf1-MBP/crRNA complex. The quantity of proteins in the sample was assessed by SDS Page electrophoresis (upper panel) and tested for DNA cleavage activity (lower panel).

Finally, we controlled that the expressed Cpf1 nucleases in the cells are functional and cleave specifically the plasmid at the target site. Because Hela cells do not allow episomal amplification of the plasmids and a high yield protein expression, we expressed AsCpf1-MBP and LbCpf1-MBP proteins in HEK 293 cells, with or without crRNA, and performed the purification using amylose resin (Figure 4A, purification of AsCpf1 protein). The purified proteins were tested for their *in vitro* cleavage activity using an *XmaI* linearized pGFP-SSA target (5′-TTTa-3′ PAM sequence) as a substrate. The purified nucleases complexes were preincubated on ice with the DNA substrate prior to the incubation at 37°C in order to observe the reaction products at the equilibrium of the Protein/DNA interaction. Using the same quantity of proteins in the reactions, the DNA cleavage at the target site was only observed with the Cpf1-MBP/crRNA purified complexes (Figure 4B: Lanes 3 and 5 versus Lanes 4 and 6). In addition, we observed that the purified LbCpf1-MBP/crRNA activity is better than the AsCpf1-MBP complex. We further tested an increasing quantity of the purified complex. The quantity of the protein was analyzed by acrylamide SDS Page (Figure 4C, top panels, 2-fold dilution cascade). We observed a difference around 10-fold in activity between the two proteins. Under the tested *in vitro* reaction conditions, we did not observe nonspecific cleavage sites of the reporter pGFP-SSA target plasmid. The *in vitro* cleavage experiment with the ribonucleo-proteinic complexes confirmed that Cpf1/crRNA are active and are able to cleave the DNA specifically at the target site. The stronger cleavage activity observed with LbCpf1-MBP/crRNA complex, compare to AsCpf1-MBP/crRNA can be associated to a better stability of the crRNA expressed in the cells, to a higher stability of the ribonucleo-proteinic complex during the purification process, or to a better catalytic activity of the complex. To observe the *in vitro* cleavage reaction in a similar condition than in a cellular context, the same reactions were performed without preincubation on ice (Supplementary Data 8) and demonstrated that the AsCpf1 nuclease cleave the plasmid DNA substrate at unspecific site compare to LbCpf1.

### Analysis of LbCpf1 Proteins PAM Selectivity Using SSA Assay

**FIGURE 5:**
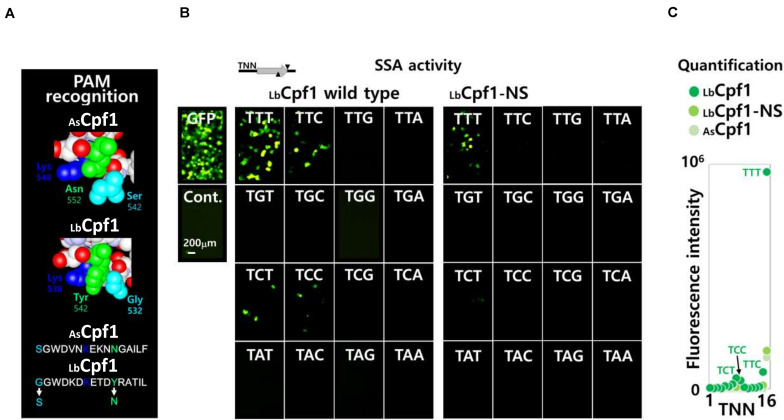
Wt and mutant LbCpf1-NS PAM selectivity analysis using a 5′-TNNa-3′ library. **(A)** Structural representation of the PAM amino acids interacting on the major groove of the DNA: representation of both enzymes AsCpf1 (S542/K548/N552) and LbCpf1 (G532/K538/Y542). The mutant LbCpf1-NS (Y532S/K542N) correspond to an amino acids swap between the two protein species depicted bellow. **(B)** Image of fluorescent cells using a fluorescent microscope (×20): Fluorescent cells were inspected after transfection and 48 h incubation. The pictures show the fluorescent cells observed by co-transfection of the crRNA with Wt or NS mutant LbCpf1, together with the different pGFP-SSA 5′-TNNa-3′ vectors. 300 ng of the Cpf1 expression vectors were transfected for this experiment. The target site, represented on top of the pictures, is the same target sequence with the 16 different 5′TNNa-3′ sequences. The GFP, and a negative control, are depicted on the left side. **(C)** Graph of the quantification (Fluorescence intensity) of the fluorescent cells with the 16 constructs pGFP-SSA 5’-TNNa-3’ for each enzyme.

Since the SSA assay show a reproducible and robust activity in Hela cells with LbCpf1, we further developed the assay to analyze the PAM selectivity of Cpf1 proteins. We generated a pGFP-SSA target plasmid library, with the same DNA target sequence, flanked with the 16 different PAM 5′-TNNa-3′ sequences. The same crRNA expressing vectors are used to analyze the cleavage activity of LbCpf1 protein and are also suitable to determine the cellular activity of the mutants. The first LbCpf1 PAM recognition mutant tested in parallel with the Wt protein (Figure 5A), is a protein with the amino acid substitutions G532S and Y542N. The lysines K538 and K595, that define the PAM sequence 5′-TTTv-3′ specificity, were not modified. As a consequence, the LbCpf1 mutant G532S/Y542N has the same PID structure than the AsCpf1 at the DNA major groove/Protein interface. To detect minor PAM interaction activities, AsCpf1 and the LbCpf1 Wt and mutant G532S/Y542N were co-transfected at high concentration (300 ng DNA), together with the crRNA expression vector, and one of the 16 pGFP-SSA 5′-TNNa-3′ target vectors. As previously observed with the Wt LbCpf1 protein, the pictures of fluorescent cells (Figure 5B) show a strong GFP expression with the PAM 5′-TTTa-3′ sequence. The presence of fluorescent cells was also observed with the pGFP-SSA target PAM 5′-TCTa-3′, 5′-TTCa-3′ and 5′-TCCa-3′ reporter constructs. The LbCpf1-NS mutant, harboring G532S and Y542N substitutions, show only a moderate GFP induction with the 5′-TTTa-3′ pGFP-SSA target construct. The fluorescent quantification for the three proteins AsCpf1, LbCpf1 and LbCpf1-NS is depicted Figure 5C. The mutant LbCpf1-NS activity is identical to the AsCpf1 protein with the PAM 5′-TNNa-3′ library.

**FIGURE 6:**
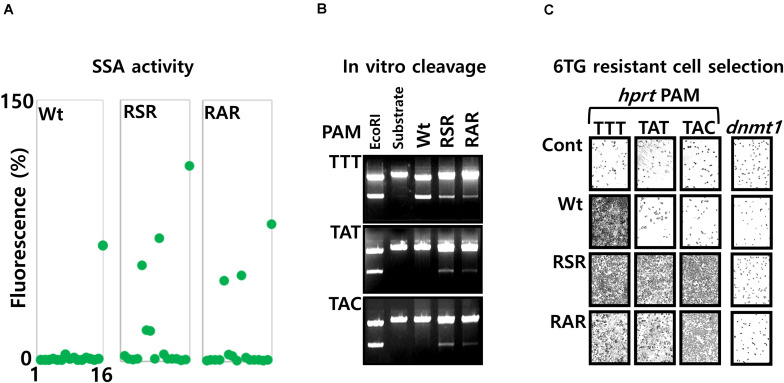
Analysis of LbCpf1 RAR and RSR mutants with the 5′-TNNa-3′ SSA library. **(A)** Graphic representation of the Wt and mutant protein activity: the graph is summarizing the activity observed with the 16 5′-TNNa-3′ reporter vectors for LbCpf1 and each PAM recognition mutant (RSR and RAR). The transfection was performed with 100 ng of LbCpf1 expression vectors. **(B)** Nuclease activity: *In vitro* cleavage activity of Wt LbCpf1-MBP and each mutants RSR-MBP and RAR-MBP with the *XmaI* linearized plasmid pGFP-SSA substrates 5′-TTTa-3′, 5′-TATa-3′ and 5′-TACa-3′. One of the plasmid substrates was digested with the *EcoRI* restriction enzyme to control the size of the products. **(C)** 6TG resistant cell selection: Pictures of Hela cells, after selection with the 6TG, following the co-transfection of the LbCpf1 (Wt or the PAM recognition mutants RSR and RAR) expression vectors, and the crRNAs that target the *hprt* exon3 sequences flanked, respectively by the 5′-TTTa-3′, 5′-TATa-3′ and 5′-TACa-3′ PAM sequences. The left Panel is a control experiment using a guide crRNA against *dnmt1* gene.

We generated further mutations, with substitution of amino acids at the position 532, 538 and 548. The mutations K538S or K538A that dramatically reduce the cellular activity (Data not shown), were associated together with the mutation Y548R. With those mutations, the negatively charged residue Lysine 538, present at the protein/DNA major groove interface of the Wt protein, is structurally replaced by an Arginine at the position 548. In addition, we substituted the amino acid Glycine 532 by an arginine to generate the two triple mutants G532R/K538S/Y548R (RSR), G532R/K538A/Y548R (RAR). The PAM sequence selectivity, analyzed with the pGFP-SSA 5′-TNNa-3′ constructs (Figure 6A), show that the mutants RSR and RAR are inducing GFP expression with the PAM sequences 5′-TTTa-3′, 5′-TATa-3′ and 5′-TACa-3′. The mutant RSR show also moderate activities with the 5′-TGCa-3′, 5′-TCCa-3′ PAM constructs.

To control the observed cellular activities, the *in vitro* cleavage assay was performed using the RSR, RAR and the Wt LbCpf1-MBP fusion enzymes with the three *XmaI* linearized target DNA pGFP-SSA plasmid substrates, containing the same target site flanked by the PAM sequences 5′-TTTa-3′, 5′-TATa-3′ and 5′-TACa-3′ (Figure 6B). As expected, the Wt LbCpf1 enzyme cleave the 5′-TTTa-3′ PAM only, whereas the mutant RSR and RAR are able to cleave the three substrates.

According to the observed recognition for their PAM sequences, the Wt LbCpf1and the mutants were tested for their efficacy to induce the modification of the *hprt* gene (exon3) at the genomic sites. Hela cells were co-transfected with the Wt or mutants LbCpf1 expression vectors, and three crRNA expression vectors targeting sequences that requires the different types of PAM sequences (5′-TTTa-3′, 5′-TATa-3′ and 5′-TACa-3′). The cells were selected with the 6TG treatment to observe the *hprt* gene disruption. The results of the 6TG resistant cells selection (Figure 6C) show the surviving cells when transfected with the PAM 5′-TTTa-3′, 5′-TATa-3′ and 5′-TACa-3′ crRNA and the RSR and RAR variants, whereas resistant cells were obtained only with the 5′-TTTa-3′crRNA when the Wt LbCpf1 is co-transfected. The guide crRNA targeting *dnmt1* gene was used as a negative control and do not allow the selection of 6TG resistant cells. We performed the same experiment using others crRNA with an upstream 5′-TATa-3′ PAM sequence, and the mutant RAR, in order to control the functionality of this mutant with several sequences (Supplementary Data 9).

## Discussion

### Nuclease Activity in a Cellular Context

In this study, we are reporting the analysis of nuclease activity in a cellular context using two complementary procedures. On the one hand, we used the deep sequencing that allow to quantify the nuclease activity at the genomic target site. This technic is measuring indels generated after NHEJ repair of the DSB generated by the nuclease. This strategy does not take into account other modification, i.e., large genomic deletion, inversion, and chromosomic translocation since they are not amplified. After genomic DNA isolation of the pool of modified cells, and PCR of a small genomic DNA region, amplified fragments are sequenced individually. On the other hand, the SSA reaction using a plasmid cleaved by the nuclease was developed. The DSB induce the recombination of repeated sequences and reconstitute an active reporter gene. This second strategy require a highly specific and selective nuclease. Indeed, the lack of nuclease specificity and selectivity leads to false negative results and wrong quantifications because of the plasmid degradation and the subsequent GFP signal destabilization. The advantage of the plasmid strategy is that the target site can be customized compare to a genomic target. In addition, the accessibility of the target site is identical compare to the variable chromatin states at genomic sites (i.e.: Euchromatin and Heterochromatin). We also favored the SSA assay with a plasmid, because it is difficult and fastidious to perform an equivalent engineering of a cell line with several plasmid constructs. Performing the SSA experiment with a stable and genomic insertion of the target gene is very interesting, however the GFP signal stability assay is not suitable. Indeed, only a *gfp* specific target sequence can disrupt the *gfp* gene inserted in the genome, and the time required (5 days) to remove the GFP protein from the cells is longer than the incubation time to induce the reporter gene. As a matter in fact, non-specific nucleases might also generate false negative samples and wrong quantification with the genomic SSA assay, by generating large genomic deletion, inversion, and chromosomic translocation at the tested locus.

In the analysis of the two Cpf1 enzymes tested using the SSA assay in this study, we showed that LbCpf1 is the nuclease with the most robust SSA cellular activity. Using the same guide RNA sequence with the two orthologue enzymes, that need the same optimal PAM sequence (5′-TTTv-3′), we observed a strong SSA activity and a low GFP signal destabilization with LbCpf1 (Supplementary Data 5B, GFP signal stability and cell surviving). This demonstrates the great selectivity of this enzyme in the cellular assay, at least for the crRNAs against *hprt* (exon 3) used in this study (Supplementary Data 6, SSA activity of 16 crRNA target sequence with LbCpf1 protein). We also observed, with the purified Cpf1-MBP/crRNA complexes, that the LbCpf1 enzyme expressed in mammalian cells has a higher *in vitro* DNA cleavage activity than AsCpf1. In addition, we demonstrated that AsCpf1 has an unspecific *in vitro* cleavage activity on the plasmid substrate without preincubation of the nuclease with the DNA on ice (Supplementary Data 8). Those observations correlate with the SSA activity of both enzymes. In this study, we performed the cellular assays, and *in vitro* cleavage assay, using Cpf1 proteins expressed from mammalian cells. Those experiments are physiologically equivalent (i.e.: proteins were exposed to the same biological environment and the potential post translational modifications associate to it). Increasing AsCpf1 expression in the SSA experiment do not lead to a better cellular activity of this protein because of the GFP destabilization associated to the non-specific cleavage activity of this enzyme observed *in vitro*. The FnCpf1 nuclease was also analyzed (Data not shown) and exerted a low activity (Zetsche et al., 2015). It was clearly demonstrated that this member of the Cpf1 nuclease family has an increased cellular activity and detectable at a genomic site by alteration (mutation RVR) of its PAM (5′-TTn-3′) recognition (Tóth et al., 2018). In addition, we also demonstrated that the nuclease spCas9 exert the same properties than AsCpf1 in a cellular context (Supplementary Data 8), and the usage of a tandem inverted repeated target site is required to obtain an efficient SSA activity without GFP signal destabilization, demonstrating the suitability of the assay to analyze other CRISPR nucleases.

The experiments conducted with the pGFP-SSA 5′-TNNa-3′ PAM sequences clearly confirmed the specificity of the Cpf1 proteins for the PAM 5′-TTTa-3′ sequence, as it was already described (Komor et al., 2016). In fact, the single change of a nucleotide, in the PAM sequence, dramatically reduced or completely abolish the cellular activity. This in turn, demonstrate that the protein must interact with the PAM sequence, prior the hybridization of the guide RNA to the target DNA sequence. This observation was also demonstrated *in vitro* using recombinant enzyme (Kim et al., 2016). Since the amino acids interacting with the PAM sequence is a small region of interaction, a single change at the protein/DNA interface dramatically reduce the affinity, and subsequently the nuclease activity in a cellular context.

Finally, we analyzed genetic modification in mammalian cells, induced by the Wt and the mutants RSR and RAR LbCpf1 proteins. We tested the targeting of the *hprt* gene and the positive selection of resistant cells upon 6TG treatment. The result of this cellular assay with the LbCpf1 Wt and the mutant correlates with the observation of the SSA experiments (PAM 5′-TTTa-3′, 5′-TATa-3′ and 5′-TACa-3′ crRNA). Altogether, our observations are reflecting that LbCpf1 nucleases used with an optimal guide RNA is sufficiently specific and selective to perform a gene disruption in mammalian cells. Nevertheless, selecting the best guide crRNA and studying its off-target effects (Kim et al., 2015; Kanchiswamy et al., 2016; Yan et al., 2017) are required to optimize the genome engineering of a target gene (Strohkendl et al., 2018).

### PAM Recognition Mutant Analysis

Since LbCpf1 and AsCpf1 are different in their PAM sequence selectivity, we first investigated the effect of a swap, between AsCpf1 and LbCpf1 amino acids. We mutagenized LbCpf1 amino acids that are different, compare to AsCpf1, on the DNA major groove/Protein interface. The mutant LbCpf1-NS is reacting as the AsCpf1 protein in the pGFP-SSA PAM 5′-TNNa-3′ selectivity assay. The fact that this amino acid swap is sufficient to modified LbCpf1 protein properties, to an AsCpf1, is supporting the central role of the PAM specificity and selectivity for the PAM sequence in the cellular activity.

Based on the observations of this study, and the mutant RR and RVR described so far with AsCpf1 protein (Gao et al., 2017), we designed further mutations of the amino acids responsible of the PAM sequence recognition in the LbCpf1 protein. We investigated the interaction with similar amino acid combination at the protein/major groove DNA interface. We substituted the lysine 538 that is directly in contact with the second Adenine of the 5′-TTTv-3′ sequence, by a Serine or an Alanine residue. We structurally replaced this positively charged residue with two Arginines at position 548 and 532. We observed that both substitutions, corresponding to the R532/A538/R548 (RAR) and R532/S538/R548 (RSR), still interact with the 5′-TTTa-3′ PAM, and allows the interaction with 5′-TATa-3′ and 5′-TACa-3′ sequences as well. Other mutant combinations were tested, such as G532/S538/E548 or E532/S538/E548 but failed to interact with all 5′-TNNa-3′ sequences tested (data not shown). In comparison, the RVR mutant described for AsCpf1 (Nishimasu et al., 2017) interacts mainly with the 5′-TATa-3′ sequences. The Valine at the position corresponding to the Serine 542 in AsCpf1 is more restrictive in the sequence selectivity, most probably because of the size of the isopropyl group of this amino acid. The modification of the second lysine K585 interacting with the PAM sequence on minor groove side of the DNA is crucial for the interaction. As with AsCpf1 (Mutant K607A), substitution of the Lysine 585 by an alanine residue, for the wild type and mutants, dramatically reduce the protein activity (Data not shown).

### PAM Specificity and Selectivity of Wt and Mutant LbCpf1

The PAM selectivity of Cpf1 proteins was demonstrated using *in vitro* experimental procedures (Komor et al., 2016), bacterial assay (Kim et al., 2017) and lentiviral expression in mammalian cells (Yamano et al., 2017). A high affinity for the 5′-TTTv-3′ sequence was reported as an optimal sequence for the two nucleases AsCpf1 and LbCpf1. The technics reported are covering larger adjacent sequences libraries and an analysis using statistical methods. Because of the number of plasmids required in both assays tested in this study, we did not cover the full diversity of the 5′ flanked region. We restricted our analysis to the 16 PAM sequences corresponding to 5′-TNNa-3′. The results of both strategies demonstrate a stringent activity for the 5′-TTTa-3′ PAM sequence with AsCpf1. On the contrary, LbCpf1 show a lower selectivity. Indeed, in addition to the high activity observed with the 5′-TTTa-3′ sequence, minor activities were observed with the PAM sequences 5′-TCTa-3′, 5′-TTCa-3′ and 5′-TCCa-3′ using both assays. This feature of LbCpf1 reflect the structural adaptability of the K538 described in the resolved structures with those particular sequences (Yamano et al., 2017). Recently, it was show that AsCpf1 interacts specifically with the PAM sequences 5′-GTT-3′ and 5′-GCT-3′ (Jacobsen et al., 2019) but we did not investigate such sequences in your study.

To be conclusive, studying independently the AsCpf1 and LbCpf1 interaction with the PAM sequences, in order to characterize and compare their respective *in vitro* affinity is required. The PID domain of the Cpf1 nucleases that interact with the PAM sequence are small regions without a distinguish structure inside the ribonucleo-proteinic complexes. To obtain a stable interaction between the PAM and the few amino acids of the PID, the guide RNA/target DNA hybridization is required. Thus, it is unfortunately not possible to isolate the PID domain and observed the interaction with the PAM sequence *in vitro*. However, it is interesting and remarkable, that despite a lower PAM selectivity than AsCpf1, LbCpf1 exert a stronger cellular activity in the SSA assay, reflecting a better selectivity for the target site. Increasing or reducing the affinity for the PAM sequences influences the cellular activity of the enzymes. A high specificity for the PAM sequence might increase unspecific effects and cleavage at more off-target sites with an upstream 5′-TTTv-3′ PAM sequence, whereas a lower affinity might reduce those undesired activities of the nucleases. Thus, a high specificity and selectivity for the PAM sequence is not required to obtain a selective nuclease for a genomic target.

### Unspecific Off-Target Effects Analysis of Nucleases

The analysis of the nuclease’s unspecific off-target effects can be addressed using assays with plasmids as demonstrated with the SSA assay and the GFP destabilization experiment. The unspecific activity of the nuclease is observed indirectly by measuring the destabilization of the GFP signal, whereas the off-target effect using deep sequencing analysis is performed on preselected sequences similar to the target site among the genome. Nevertheless, the experiments with plasmids are rapid and cost-effective assays for a primary experiment to demonstrate and characterize the nuclease activity and their unspecific off-target effects. On the contrary, the Deep sequencing is more precise to quantify the off-target activity and analyze the effects of mutations. The deep sequencing experiment is focusing the analysis at similar sites that are selected but not discreet, and do not answer a general view of unspecific activity of the nucleases. In our study, we evaluated the effects of mutations affecting the PAM recognition of LbCpf1, we tested the mutant RSR and RAR that were not evaluated to date but that are similar to the described mutations RVR (Gao et al., 2017) or RRVR (Tóth et al., 2020). The previous studies about those type of mutations demonstrated that they are not reducing the off-target effects, but they are expending the cleavage activity to the all PAM sequences accessible with similar sequences to the target. Reducing the off-target effects need further mutations affecting other domains of the protein Cpf1/RNA complex (Gao et al., 2017).

*In fine*, our results demonstrate the complementarity of all technologies developed so far, NGS analysis, SSA plasmid assay and *in vitro* DNA cleavage, to characterize the nuclease activity of CRISPR proteins. Altogether, the experimental information of those assays is useful for the genetic manipulation of eukaryotic cells.

## Data Availability Statement

The raw data supporting the conclusions of this article will be made available by the authors, without undue reservation.

## Author Contributions

VB designed and performed the experiments, analyzed and interpreted the data. US did the NGS. VB wrote the manuscript. Both authors contributed to the article and approved the submitted version.

## Conflict of Interest

The authors declare that the research was conducted in the absence of any commercial or financial relationships that could be construed as a potential conflict of interest.

## Supplementary Material

Supplementary Data 1 Sequences of the oligonucleotides cloned into the pU6 vectors for the AsCpf1 and LbCpf1crRNA expression in mammalian cells.

Supplementary Data 2 **(A)** Representation of the *hprt* exon 3 target sequence used in the disruption cellular assay. **(B)** Deep sequencing analysis of cells 6TG resistance selected after the nuclease transfection with guide RNA: Hela cells were co-transfected with LbCpf1 and the 5′-TTTa-3′ Guide RNA. A control experiment was performed with a guide RNA targeting *Dnmt1* gene. The 6TG selection was performed after 5 days incubation. Following the 6TG exposure, the genomic DNA was isolated. The PCR amplified DNA of the *hprt* gene (exon 3) was analyzed by Deep sequencing (percentage of Indels frequency). The graphic show the positions and the quantification of insertions and deletions at the *hprt* target site

Supplementary Data 3 **(A)** Vector map of the pGFP-SSA reporter plasmid. **(B)** Sequence of SSA *gfp* fragments: the 5′ and 3′ sequences of the *gfp* gene fragments are depicted, the dark green sequences are corresponding to the left and right repeat that recombine during the HR reaction.

Supplementary Data 4 Sequences of the oligonucleotides cloned between *EcoRI* and *BamHI* in the pGFP-SSA reporter plasmid. Sequences of the oligonucleotides used for the mutagenesis of the LbCpf1 expression plasmid.

Supplementary Data 5 **(A)** Control of pGFP-SSA activity in transfected Hela cells: Hela cells were transfected with the pGFP positive control and the circular, or *EcoRI* linearized, pGFP-SSA reporter plasmids. The pictures show the fluorescent cells using a fluorescent microscope (×20) after transfection and 48 h incubation. The *EcoRI* linearized pGFP-SSA vector was used as a control for samples normalization of the SSA assay. **(B)** GFP signal stability and cell surviving: Hela cells were transfected with the pGFP control expression vector and the Cpf1 proteins with the guide RNA against the same target sequence (*hprt* gene, Exon 3, 5′-TTTa-3′ PAM sequence). The cells surviving and the GFP fluorescence were quantified after 48 h of incubation. The results indicate that the cells survived to Cpf1 expression, but the GFP signal is lower with AsCpf1 compare to LbCpf1.

Supplementary Data 6 SSA activity of 16 crRNA target sequence with LbCpf1 protein: The 16 target sequences, used for the deep sequencing analysis in Figure 2, were cloned in pGFP-SSA reporter vector. All target sequences are flanked by the optimal 5′-TTTa-3′ PAM sequence, instead of the genomic PAM 5′-TNNa-3′ sequences. The 16 pGFP-SSA 5′-TTTa-3′ target vectors were transfected with the corresponding guide crRNA expression plasmids and the LbCpf1 expression vector at 300 ng. Fluorescent cells were quantified after 48 h of incubation and reported in the graph as a percentage of fluorescence, the calculation was made using *EcoRI* linearized pGFP-SSA as a reference. A negative control was performed with the empty pGFP-SSA vector.

Supplementary Data 7 Analysis of spCas9 with the SSA assay. **(A)** Representation of the *hprt* exon 3 target sequence used in the disruption cellular assay with SpCas9 nuclease and analysis by deep sequencing of the cells resistant to the 6TG: The Hela cells were selected after the transfection of the plasmid expressing the nuclease and the guide RNA. A control experiment was performed with a guide RNA targeting *Dnmt1* gene. The graphic show the positions and the quantification of insertions and the deletions at the *hprt* target site (percentage of Indels frequency). **(B)** GFP signal stability: The unspecific off-target effects of the Wt SpCas9, the Nickase (N-Cas9: single mutant D10A) and the catalytically inactivated nuclease (D-Cas9, termed dead-Cas9: double mutant D10A/H840A) is tested by co-transfection of the plasmids expressing SpCAS9 (500 ng) and the guide RNA with the pGFP expression vector. The Wt SpCas9 nuclease show a low GFP signal compare to the nickase and the dead nuclease demonstrating the destabilization of the GFP signal. C. SSA activity of SpCas9 nuclease: The activities of the Wt spCas9, N-Cas9 and D-Cas9 proteins with the SSA assay were tested. The Hela cells were co-transfected with the plasmids expressing the proteins, the guide RNA and the pGFP-SSA reporter vectors containing the corresponding target site and an inverted tandem repeat. The two reporters allow to observe the differential activity of the Wt SpCas9 and the mutant N-Cas9 protein. As expected, the catalytically inactivated nuclease and the Nickase are inactive on the target site. The SpCas9 nuclease is active on both SSA reporter constructs whereas N-Cas9 is active on the tandem repeats only.

Supplementary Data 8 *In vitro* cleavage activity of the Cpf1 nucleases without pre-incubation on ice. **(A)** Schematic representation of the Cpf1 nuclease interactions with the specific target and the off-target site on both conditions, with and without preincubation on ice. **(B)**. *In vitro* activity of Cpf1-MBP purified proteins without preincubation on ice: The cleavage activity of Cpf1-MBP proteins expressed with the crRNA was performed with pGFP-SSA 5′-TTTa-3′ linearized *XmaI* target plasmid. The unspecific cleaved product observed with AsCpf1 is depicted (^∗^) on the side of picture. The substrate was digested with the restriction enzyme *EcoRI* to control the size of the specific products after cleavage.

Supplementary Data 9 Selection of 6TG resistant cells with the RAR mutant: **(A)** Target sequences: The sequences of the crRNA with a flanked 5′-TATa-3′ PAM within the target *hprt* gene (exon3) are depicted. **(B)** Quantification of the 6TG resistant cells: The graph represent the results of the luminescence observed by cell titer Glo quantification, after selection with the 6TG, following the co-transfection of the LbCpf1 (Wt or the PAM recognition mutant RAR) expression vectors, and the crRNAs that target the *hprt* exon3 sequences flanked by the 5′-TATa-3′ PAM sequence.

## Abbreviations

6-TG: 6-Thioguanine
As: Acidaminococcus
Cas: CRISPR-associated protein
CMV: early enhancer/chicken β actin promoter
Cpf1: CRISPR from Prevotella and Francisella 1
CRISPR: Clustered regularly interspaced short palindromic repeat
crRNA: CRISPR RNA
DMEM: Dulbecco’s modified Eagle Medium
DMSO: Dimethyl sulfoxide
DNMT: DNA methyltransferase
DSB: Double strand Break
Fn: Francisella novicida
FokI: Flavobacterium okeanokoites
GFP: Green fluorescent protein
HPRT: Hypoxanthine Phosphoribosyltransferase
HR: Homologous recombination
Indels: Insertion- deletion
Lb: Lachnospiraceae bacterium
MBP: Maltose binding protein
NHEJ: Non-homologous end joining
PAM: Protospacer adjacent motif
sgRNA: single guide RNA
Sp: Streptococcus pyogenes
SSA: Single strand annealing
TALEN: transcription activator-like effector nucleases
tracrRNA: Transactivating CRISPR RNA
Wt: wild type
ZFN: Zinc finger nuclease.
