# Supplementary figures and images for: Analysis of Wild Type LbCpf1 Protein, and PAM Recognition Variants, in a Cellular Context
Source: Front Genet. 2021 Jan 7;11:571591. doi: 10.3389/fgene.2020.571591 (PMC7817983; doi:10.3389/fgene.2020.571591)

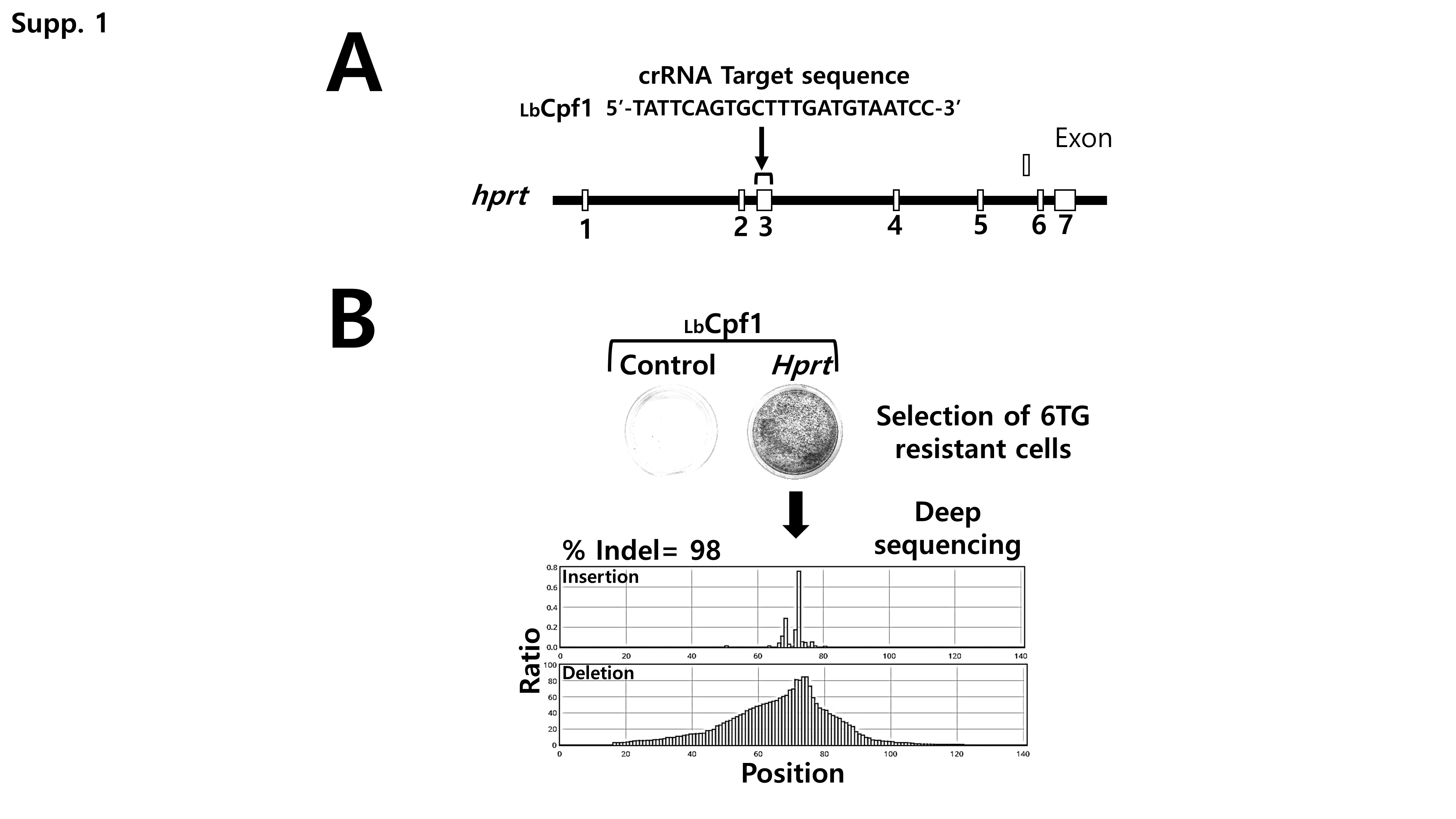

Supplement: Supplementary Data 1 — Sequences of the oligonucleotides cloned into the pU6 vectors for the AsCpf1 and LbCpf1crRNA expression in mammalian cells. [file Image_1.TIF]

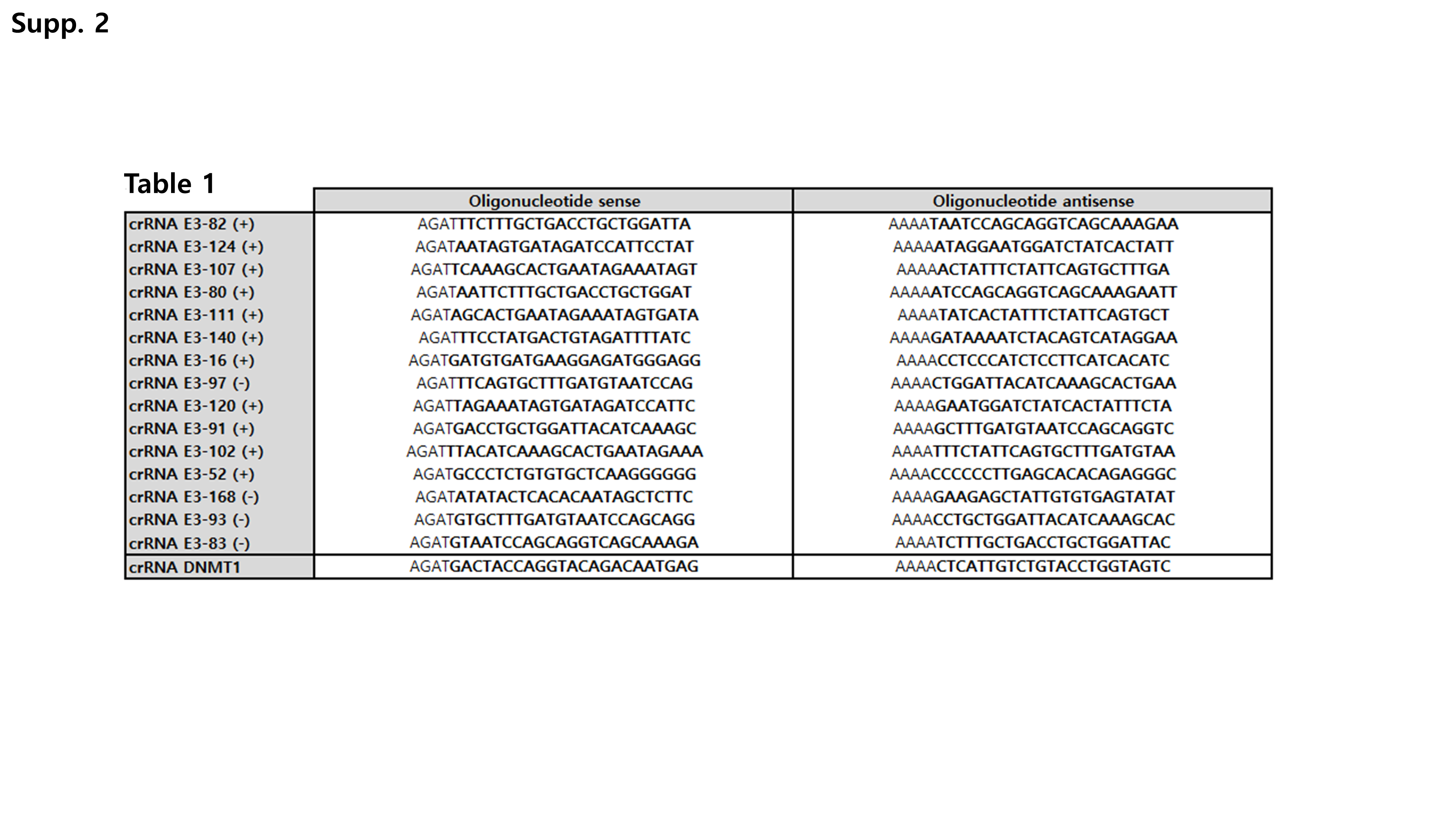

Supplement: Supplementary Data 2 — (A) Representation of the hprt exon 3 target sequence used in the disruption cellular assay. (B) Deep sequencing analysis of cells 6TG resistance selected after the nuclease transfection with guide RNA: Hela cells were co-transfected with LbCpf1 and the 5′-TTTa-3′ Guide RNA. A control experiment was performed with a guide RNA targeting Dnmt1 gene. The 6TG selection was performed after 5 days incubation. Following the 6TG exposure, the genomic DNA was isolated. The PCR amplified DNA of the hprt gene (exon 3) was analyzed by Deep sequencing (percentage of Indels frequency). The graphic show the positions and the quantification of insertions and deletions at the hprt target site [file Image_2.TIF]

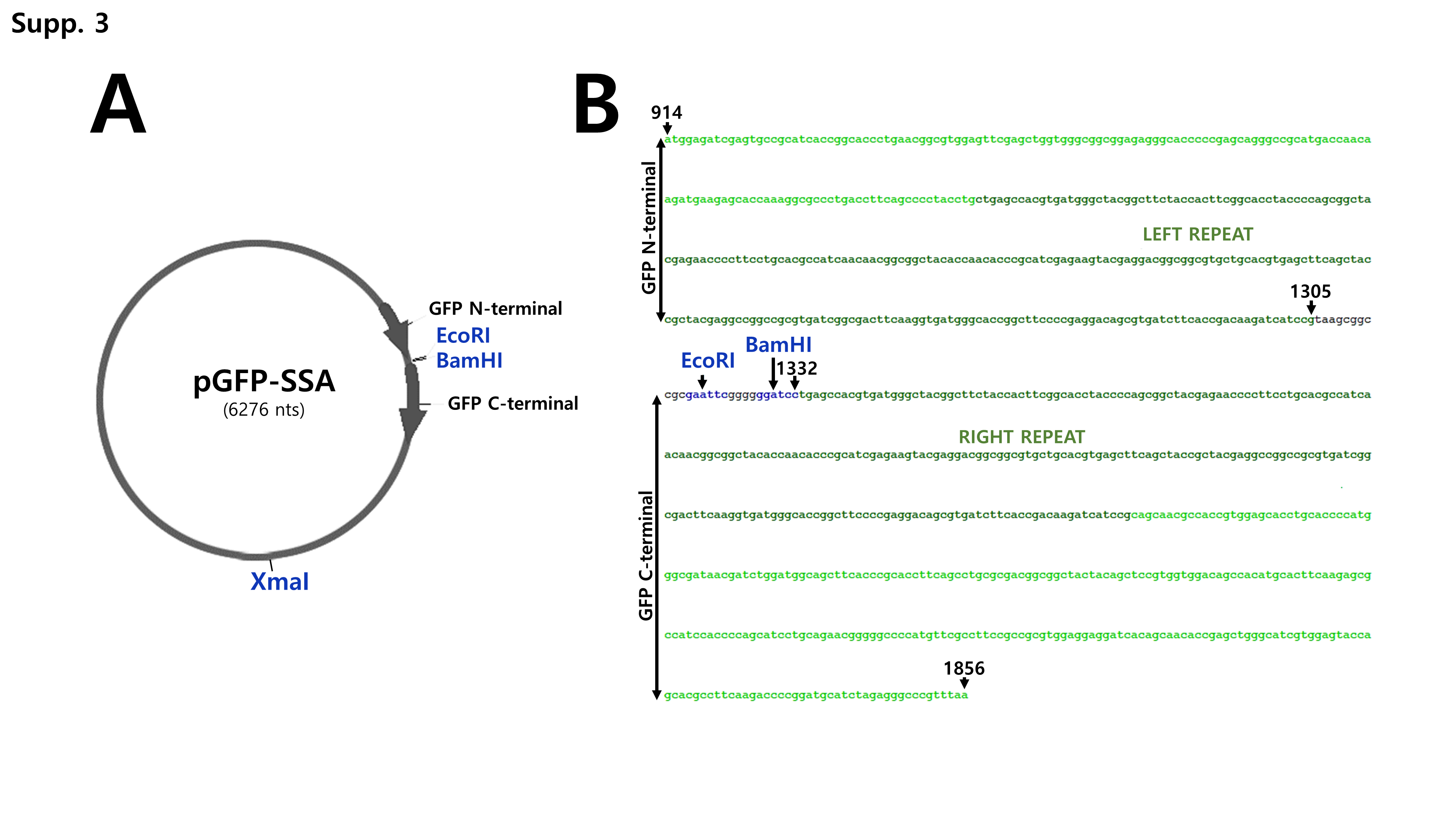

Supplement: Supplementary Data 3 — (A) Vector map of the pGFP-SSA reporter plasmid. (B) Sequence of SSA gfp fragments: the 5′ and 3′ sequences of the gfp gene fragments are depicted, the dark green sequences are corresponding to the left and right repeat that recombine during the HR reaction. [file Image_3.TIF]

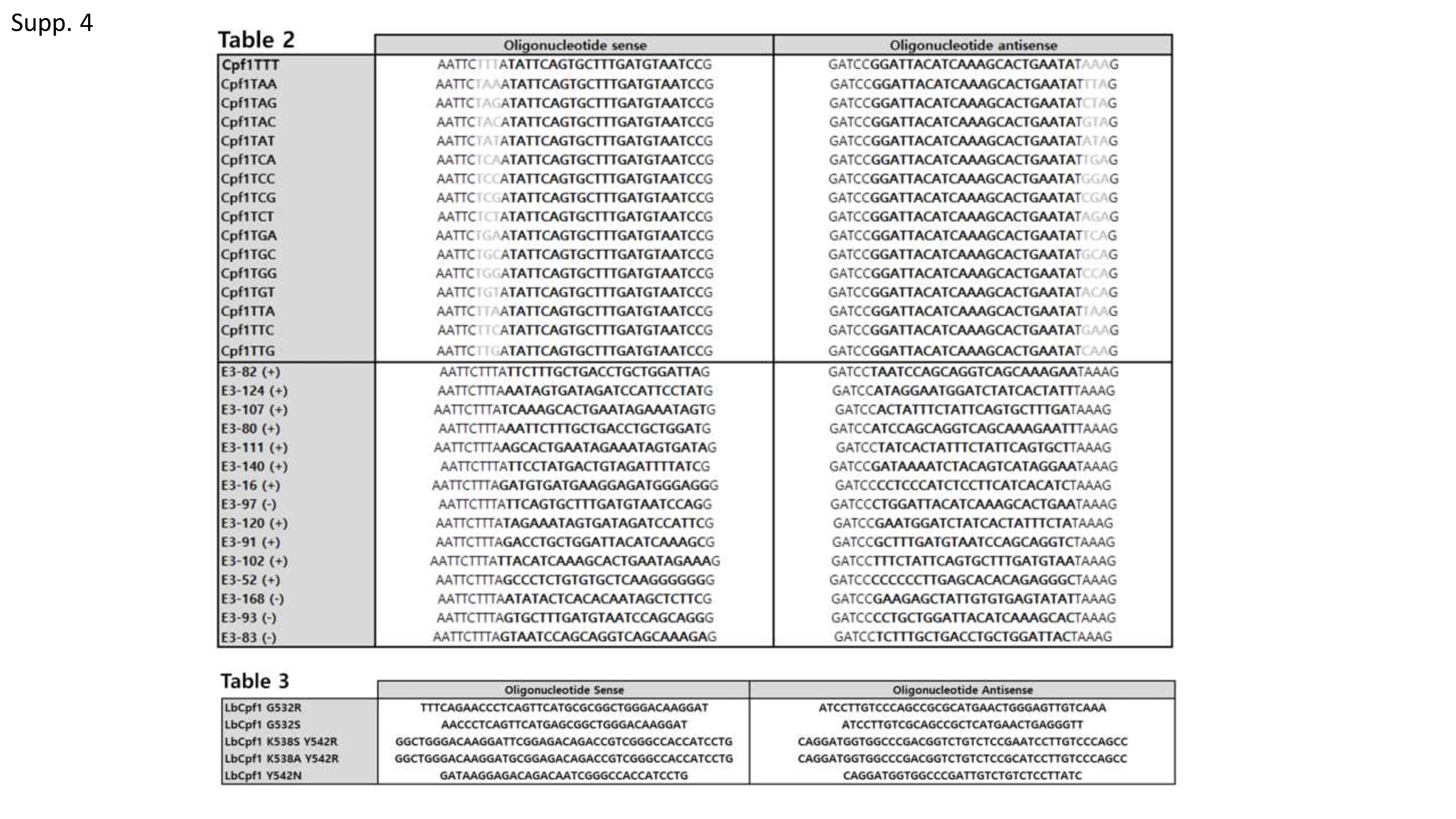

Supplement: Supplementary Data 4 — Sequences of the oligonucleotides cloned between EcoRI and BamHI in the pGFP-SSA reporter plasmid. Sequences of the oligonucleotides used for the mutagenesis of the LbCpf1 expression plasmid. [file Image_4.TIFF]

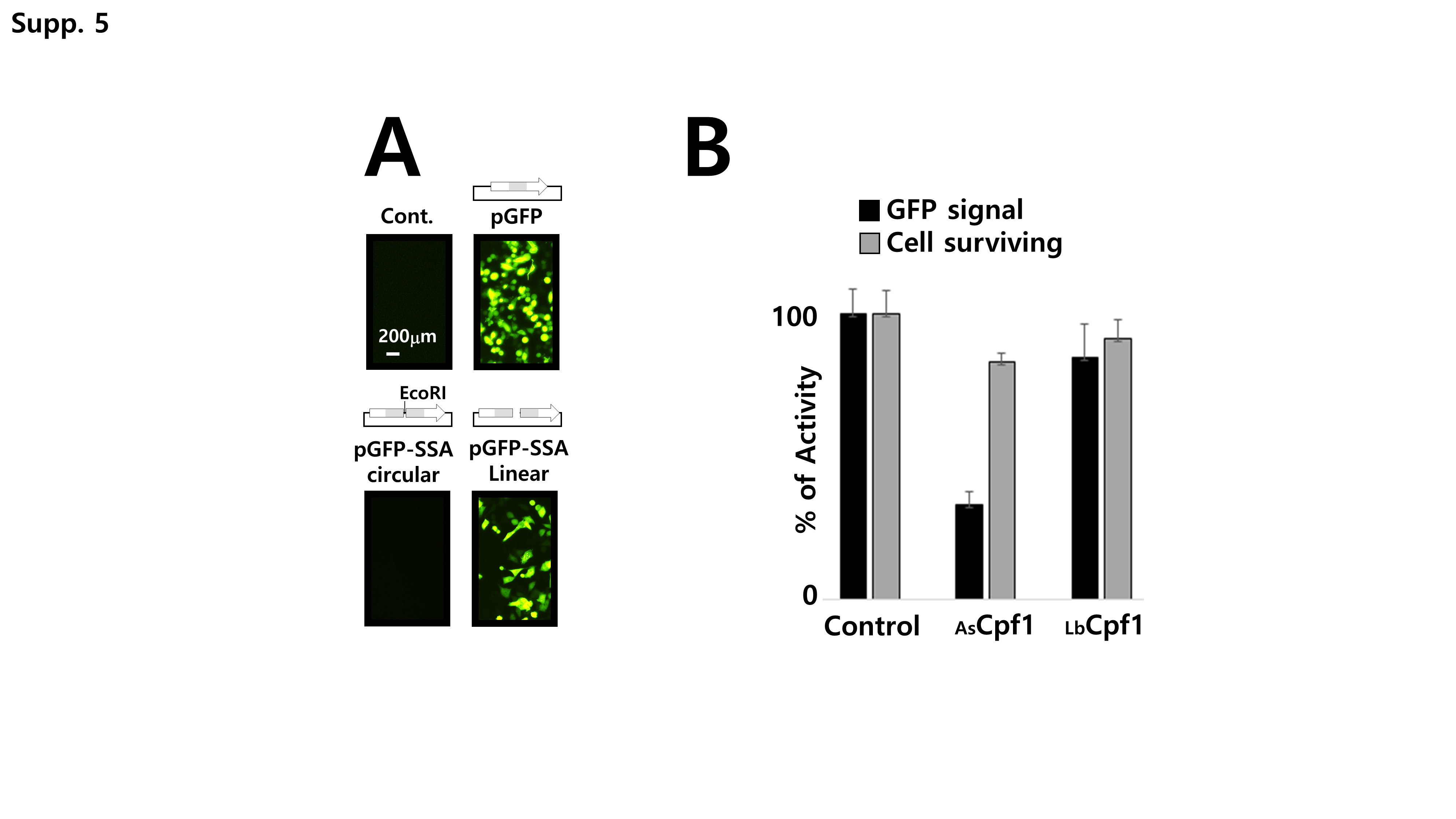

Supplement: Supplementary Data 5 — (A) Control of pGFP-SSA activity in transfected Hela cells: Hela cells were transfected with the pGFP positive control and the circular, or EcoRI linearized, pGFP-SSA reporter plasmids. The pictures show the fluorescent cells using a fluorescent microscope (×20) after transfection and 48 h incubation. The EcoRI linearized pGFP-SSA vector was used as a control for samples normalization of the SSA assay. (B) GFP signal stability and cell surviving: Hela cells were transfected with the pGFP control expression vector and the Cpf1 proteins with the guide RNA against the same target sequence (hprt gene, Exon 3, 5′-TTTa-3′ PAM sequence). The cells surviving and the GFP fluorescence were quantified after 48 h of incubation. The results indicate that the cells survived to Cpf1 expression, but the GFP signal is lower with AsCpf1 compare to LbCpf1. [file Image_5.TIF]

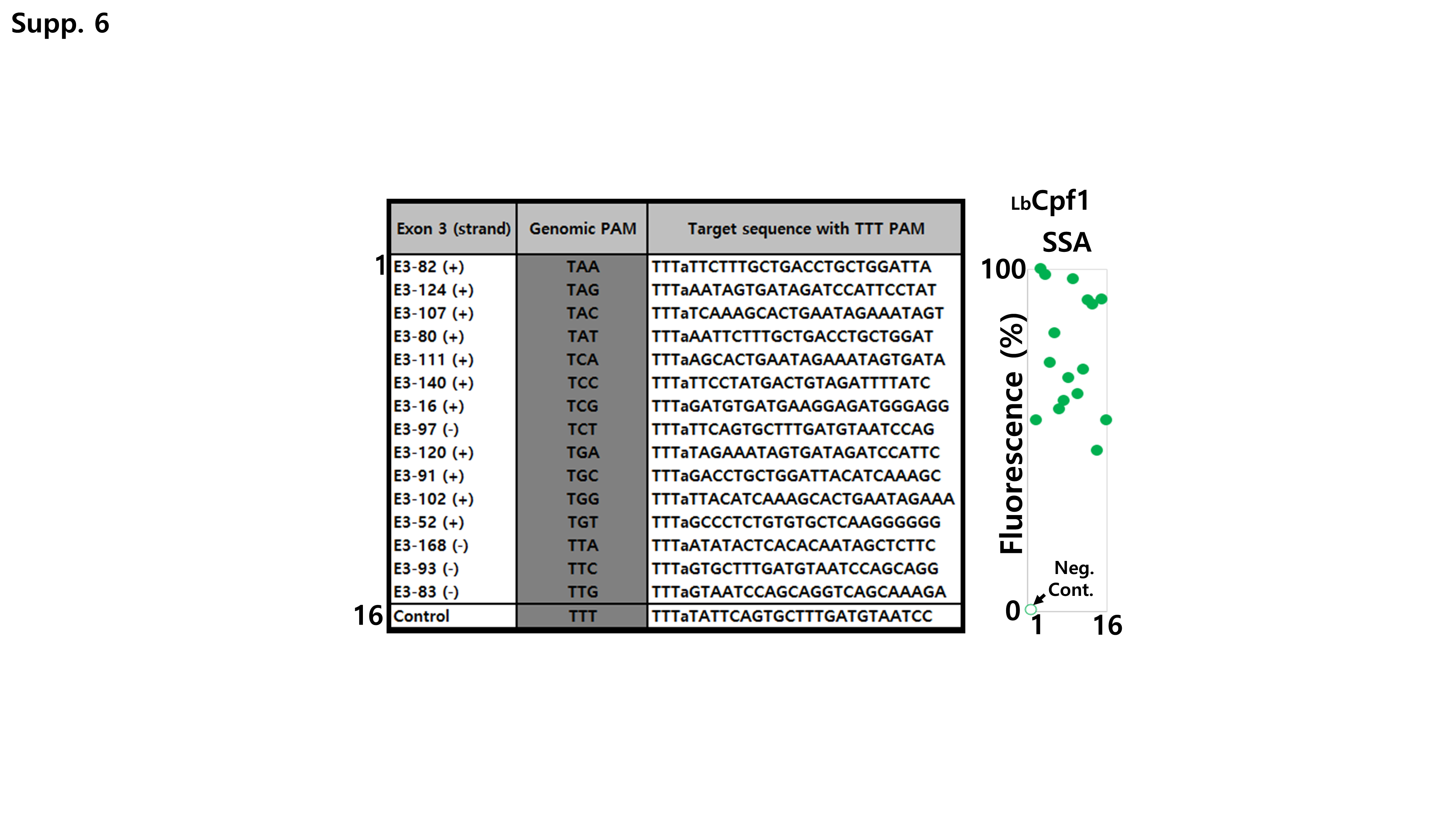

Supplement: Supplementary Data 6 — SSA activity of 16 crRNA target sequence with LbCpf1 protein: The 16 target sequences, used for the deep sequencing analysis in Figure 2, were cloned in pGFP-SSA reporter vector. All target sequences are flanked by the optimal 5′-TTTa-3′ PAM sequence, instead of the genomic PAM 5′-TNNa-3′ sequences. The 16 pGFP-SSA 5′-TTTa-3′ target vectors were transfected with the corresponding guide crRNA expression plasmids and the LbCpf1 expression vector at 300 ng. Fluorescent cells were quantified after 48 h of incubation and reported in the graph as a percentage of fluorescence, the calculation was made using EcoRI linearized pGFP-SSA as a reference. A negative control was performed with the empty pGFP-SSA vector. [file Image_6.TIF]

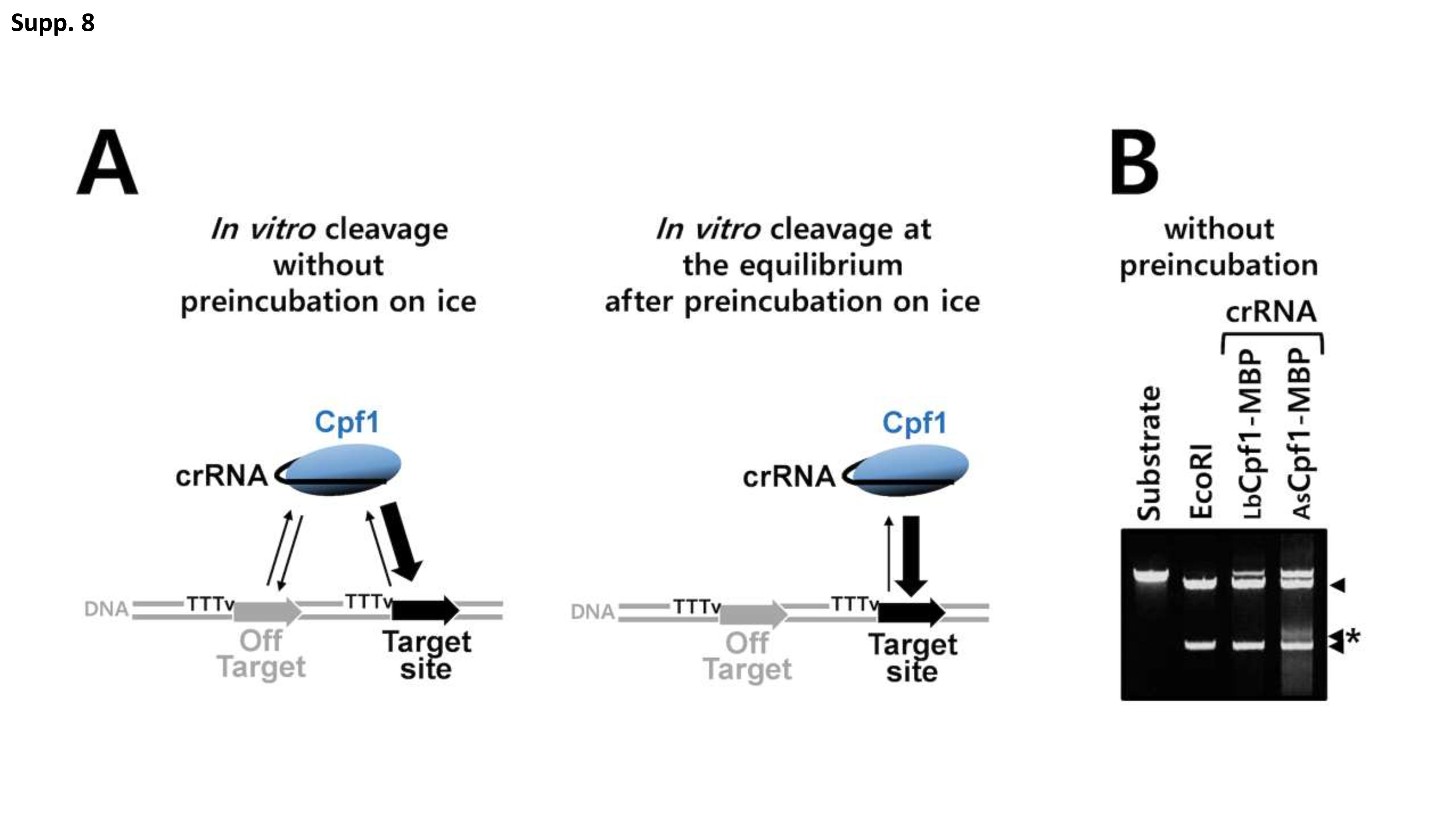

Supplement: Supplementary Data 8 — In vitro cleavage activity of the Cpf1 nucleases without pre-incubation on ice. (A) Schematic representation of the Cpf1 nuclease interactions with the specific target and the off-target site on both conditions, with and without preincubation on ice. (B). In vitro activity of Cpf1-MBP purified proteins without preincubation on ice: The cleavage activity of Cpf1-MBP proteins expressed with the crRNA was performed with pGFP-SSA 5′-TTTa-3′ linearized XmaI target plasmid. The unspecific cleaved product observed with AsCpf1 is depicted (∗) on the side of picture. The substrate was digested with the restriction enzyme EcoRI to control the size of the specific products after cleavage. [file Image_8.TIFF]
